# The Relationship Between Self-Efficacy and Aggressive Behavior in Boxers: The Mediating Role of Self-Control

**DOI:** 10.3389/fpsyg.2019.00212

**Published:** 2019-02-19

**Authors:** Xin Chen, Guodong Zhang, Xueqin Yin, Yun Li, Guikang Cao, Carlos Gutiérrez-García, Liya Guo

**Affiliations:** ^1^College of Physical Education, Institute of Sports Science, and Key Lab of Physical Fitness Evaluation and Motor Function Monitoring of General Administration of Sports of China, Southwest University, Chongqing, China; ^2^Center for Mental Health Education, School of Psychology, Southwest University, Chongqing, China; ^3^Department of Physical and Sport Education, Universidad de León, León, Spain

**Keywords:** self-efficacy, self-control, aggressive behavior, mediating effect, boxer

## Abstract

Aggressive behavior has been one of the core issues in sports psychology, whereas boxers’ aggressive behavior has received limited attention. Although some literature reported that self-efficacy is related to aggressive behavior, the mechanism whereby self-efficacy affects aggressive behavior remains unclear. The present study investigated the relationship between self-efficacy and aggressive behavior, as well as the effect of self-control as a mediating factor. This study uses the Self-efficacy Scale for Athletes, the Self-control Questionnaire for Athletes, and the Buss-Perry Aggression Questionnaire. This relationship is explored through self-reported measures from *N* = 414 Chinese professional boxers, *n* = 243 were male and *n* = 171 were female, the average age was *M* = 17.72 years (SD = 3.147), the participants, the average number of years of exercise was *M* = 3.89 years (SD = 2.734); Results showed that male boxers reported greater aggression than female boxers; It was found that the self-efficacy and self-control improved as age of the participants increased; The higher the level of competition, the higher levels of self-efficacy and self-control; Self-efficacy was negatively related with aggressive behavior and positively correlated with self-control. Self-control was also negatively correlated with aggressive behavior among boxers. Self-control had a full mediating effect on the relationship between self-efficacy and aggressive behavior.

## Introduction

Athletes abide by the Olympic motto, “faster, higher, stronger” in their arduous training and competitions, and they strive to maintain an optimal performance state (United States Olympic Committee [USOC], 1993). While rigorously improving themselves and pursuing optimal performance, they may encounter various psychological problems. Aggressive behavior in athletes is one of the core issues concerning the *International Society of Sports Psychology* (Tenenbaum et al., 1997). Most studies have shown that there is a significant negative correlation between aggressive behavior and sports performance (McCaw and Walker, 1999; Shafizadeh, 2008). Most of the existing research focused on ordinary people’s aggressive behavior; and the research on aggressive behaviors in sporting contexts focused on Football athletes (Krenn and Meier, 2018), Basketball athletes (Carleton et al., 2016), Rugby athletes (Kerr, 2018), Ice hockey athletes (Cusimano et al., 2016), Baseball athletes (Krenzer and Splan, 2018), etc. However, there is a lack of attention to the aggressive behavior of combat athletes, such as Boxer, Kickboxing, Muay Thai, etc. Furthermore, unlike group ball sports, Boxing is a combat sport that places two individuals in intense one-on-one physical and mental competition where physical injury or even death (British Medical Association Scientific Department, 1993) is a distinct possibility. Therefore, this study focuses on aggressive behavior of boxers, and investigates whether there are differences between different levels of boxers’ aggressive behaviors.

In the social learning theory, Bandura (1973) attributed aggressive behavior to a wide variety of social phenomena. Anderson and Bushman (2002) defined aggressive behavior as exerting intentionally inflicts substantial harm to another individual, and the formation of aggressive behavior is a cognitive process in which personal and environmental factors influence each other. In the field of sports, previous research suggests that malicious aggressive behavior in sporting competitions can lead to anti-social behavior in athletes (Rowe, 1998; Kavussanu et al., 2013). As such, aggressive behavior remains to be one of the most critical issues in many disciplines. Bandura (1997) found that reducing aggression and violence in adolescents requires very strong self-efficacy, and most researchers believe that self-efficacy can protect the physical and mental health of individuals (Benight and Bandura, 2004; Luszczynska et al., 2009). Self-efficacy is defined by Bandura (1977, 1986) as the degree of one’s feelings about one’s ability to accomplish goals. Some studies have shown that self-efficacy is related to aggressive behavior (Pompili et al., 2007; Piko and Pinczés, 2014; Allen et al., 2018). Few studies have been explicitly conducted to assess the association of self-efficacy with aggressive behavior (Wu et al., 2015; Brubacher et al., 2016). Additionally, prior studies have neglected to explore the intrinsic mechanism of self-efficacy that affects aggressive behavior. Therefore, this study surveyed Chinese professional boxers to shed some light on the impact of self-efficacy on aggressive behavior and of the paths of such effects. This provides theoretical guidance for the prevention and intervention of aggressive behavior in athletes.

### Self-Efficacy and Aggressive Behavior

According to social cognitive theory, aggressive behavior is caused by the cognitive bias of the aggressor, and low expectations of one’s ability and performance may lead to anti-social behavior (Ganooverway et al., 2009). Specifically, reducing aggressive and violent behavior requires for a strong sense of self-efficacy (Bandura, 1994; Bandura, 1997). Studies have found that self-efficacy in emotion regulation effectively regulated the externalization of aggression and criminal behavior, and that self-efficacy in emotion regulation was negatively related with aggressive behavior (Caprara et al., 2010; e.g., Wu et al., 2015). In addition, studies in different research areas have shown that self-efficacy predicted aggressive behavior (Buser et al., 2015). Particularly, in sports, researchers have found that self-efficacy acted as a psychological mechanism that triggers aggressive behavior and that the use of exercise intervention may improve self-efficacy and then reduce aggressive behavior (Khademi Mofrad and Mehrabi, 2015). In other words, an increase in self-efficacy resulted in a decrease of aggression (e.g., Buser et al., 2015). The sense of self-efficacy in sports refers to the individual’s belief of sports ability, and it is the individual’s assessment about whether he or she could use his or her own abilities or skills to complete the tasks in sports, in other words, it can refers to a subjective judgment that controls one’s sports behaviors and competence (Wei et al., 2008). Based on the considerations aforementioned, we assume that self-efficacy helps athletes to deal with negative emotions effectively and to restore the balance of physical and psychological states, subsequently inhibiting aggressive behavior: Boxers with higher self-efficacy should display lower aggression levels. Thus, the first hypothesis (H1) states that self-efficacy is a significant negative predictor of aggressive behavior.

### Self-Control and Self-Efficacy

Self-regulation theory proposes that self-control is influenced by self-efficacy (e.g., Bandura, 1977). In general, self-efficacy is an important factor that influences an individual’s ability to exert self-control and it can further explain the ability to exhibit self-control (Graham and Bray, 2015). Baumeister (2002) found that self-control depends on an individual’s self-control resources, while self-efficacy complements such resources by acting as a positive emotion. Numerous studies have shown a positive association between self-efficacy and self-control, and that self-efficacy can significantly predict self-control (Hamedani, 2013; Au, 2015; Huang and Yang, 2015). In sports, the theory of challenge and threat posits that under a challenging situation, athletes may exhibit a higher self-efficacy and sense of control, hold approach achievement goals, and perform better in competitions (Jones et al., 2009). On the one hand, Chinese researchers have found that self-efficacy and self-control in athletes influence each other (Li et al., 2013). On the other hand, in boxing, high self-efficacy and high self-control ability are prerequisites for boxers to excel (Ba and Zhu, 2008). Athletes’ self-control refers to the individuals’ ability to overcome or change internal reactions, suppress impulses, and interrupt impulsive behavior response trends, such as changing and adjusting behaviors, thoughts, emotions, and habituation (Li and Zhang, 2011). So, does a boxer’s self-efficacy have a positive effect on self-control? There are currently limited academic research results on this. Therefore, our second hypothesis (H_2_) states that self-efficacy and self-control of boxers are positively correlated.

### Self-Control and Aggressive Behavior

Several studies have suggested the potential role of self-control in aggressive behavior (DeWall et al., 2011). Self-control can improve the ability to adapt to the living environment (Coyne and Wright, 2014). Most researchers have found a significant negative correlation between self-control and aggressive behavior (White and Turner, 2014; Pung et al., 2015; Meldrum et al., 2016). They have also found that self-control training can reduce an individual’s level of aggression (Denson, 2013; Duckworth et al., 2014; Wang et al., 2017). Researchers have observed that individuals with reduced self-control were likely to exhibit more aggressive behavior (Teng et al., 2014; Osgood and Muraven, 2016). In sports, self-control plays a critical role in the performance of athletes and is decisive in the control of aggressive behavior (Dorris et al., 2012). However, mixed findings also existed. For example, it has been found that individuals who engage in self-control display greater aggression than those who do not (Dewall et al., 2007; Stucke and Baumeister, 2010). The general theory of crime is of the view that all delinquencies and problem behaviors are due to low self-control (Gottfredson and Hirschi, 1990). By improving the internal psychological characteristics of self-control, individuals can reduce aggressive and problem behaviors (e.g., Gottfredson and Hirschi, 1990; Xin et al., 2007). Several research have shown that among different groups, self-control played a mediating role between other psychological traits and aggressive behavior (Li et al., 2017; Song et al., 2017; e.g., White and Turner, 2014; Zhang, 2016). Therefore, there is still a lack of research on the relationship between self-control and aggressive behavior in sports; does self-control also play a mediating role between self-efficacy and aggressive behavior in boxers? In view of the lack of research on the interactions between self-efficacy, self-control, and aggressive behavior, Based on the above literature, this study proposed our hypothesis H3 and H4: Self-control would be negatively correlated with aggressive behavior, self-control would play a mediating role in the relationship between self-efficacy and aggressive behavior.

## Materials and Methods

### Participants

This study adopted cluster sampling and selected boxers from the Chinese national boxing team and boxing teams of Sichuan, Chongqing, Guizhou, and Yunnan provinces as participants to complete a survey questionnaire. Permission was obtained from the University’s Human Research Ethics Committee. Prior to answering the items, participants read information about the purpose of the study, implications of participation, and data protection. The information stressed that participation was completely voluntary and anonymous. A total of 450 questionnaires were distributed and *N* = 414 valid ones were returned (92% response) rate. Among the participants, *n* = 243 were male (58.7%) and *n* = 171 were female (41.3%). According to Chinese technical classification of athletes, *n* = 255 participants were classified as Level 3 athletes (54.3%), *n* = 57 as Level 2 athletes (13.8%), and *n* = 64 as Level 1 athletes (15.5%), and 68 athletes at the Master Level or above (16.4%), the higher the level in turn (Gazette of the State Council of the People’s Republic of China, 1995). Their average age was *M* = 17.72 years (SD = 3.147), and their average experience in training was *M* = 3.89 years (SD = 2.734). Sixty-nine participants were younger than 16 years. Written and informed consent was obtained from the parents/legal guardians of all non-adult participants.

### Procedure

Written and informed consent was obtained from the parents/legal guardians of all non-adult participants. The participants in this study were all national boxers who were strictly trained before the survey was conducted. After receiving informed consent from the management, coaches, and athletes of the national team and other sports teams, the questionnaire was distributed to teams at the provincial level or above. The questionnaires were distributed at the National Olympic Training Center, Beijing Sport University, Qujing in Yunnan, Qingzhen in Guizhou, Shapingba in Chongqing, and Liangshan in Sichuan between the 12th of June and the 8th of July, 2017. The instructions were explained in detail and example questions were provided to the participants, who were asked to read the questionnaire carefully and answer according to their actual circumstances. The questionnaire took about 20 min to complete.

### Measures

This study employed the surveys. Specifically, the following three questionnaires were included in the study, a total of questions that took minutes to complete by boxer participants.

### Self-Efficacy Scale for Athletes

Based on self-efficacy theory and related theories and knowledge about competitive sports, Wei et al. (2008) compiled a self-efficacy scale for athletes in heavy athletics sport. This scale included 15 items, such as “I can keep my mind clear and focused during the competition.” Each item was measured by a five-point scale (1 = never been like this; 5 = always so). A higher score indicates a higher self-efficacy. The one-dimensionality of the scale was proved by a confirmatory factor analysis (CFA): χ^2^/*df* = 1.086, RMSEA = 0.014, TLI = 0.996, GFI = 0.976, NFI = 0.969, CFI = 0.997, IFI = 0.997. The factor loadings (regression coefficients) of the items ranged from α = 0.355 to α = 0.690. The internal consistency of the scale was good (Cronbach’s α = 0.91).

### Self-Control Questionnaire for Athletes

Li and Zhang surveyed 820 professional athletes in China and created a self-control scale for athletes. Their questionnaire contains 24 items, which are scored on a five-point scale, ranging from “1 = not at all” to “5 = very much”; higher scores indicate a better self-control (item example: “In order to complete the training task, I can endure extreme fatigue.”). In a previous study, the scale provided a reference for athlete selection (e.g., Li and Zhang, 2011). A confirmatory factor analysis proved the one-dimensionality of the scale χ^2^/*df* = 1.220, RMSEA = 0.023, TLI = 0.975, GFI = 0.954, NFI = 0.910, CFI = 0.982, IFI = 0.982. The factor loadings of the items ranged between α = 0.380 and α = 0.652. The internal consistency of the scale was good (Cronbach’s α = 0.85).

### Buss-Perry Aggression Questionnaire

We used the modified Chinese version (Yang and Wang, 2012) of the Aggressive Behavior Questionnaire developed by Buss and Perry (1992) to assess adolescent aggressive behavior. The questionnaire consists of four dimensions. The physical aggression CFA results were good: χ^2^/*df* = 1.762, RMSEA = 0.043, TLI = 0.975, GFI = 0.943, NFI = 0.972, CFI = 0.987, IFI = 0.987. The verbal aggression CFA results were good: χ^2^/*df* = 1.520, RMSEA = 0.035, TLI = 0.988, GFI = 0.965, NFI = 0.986, CFI = 0.995, IFI = 0.995. The anger CFA results were good: χ^2^/*df* = 1.105, RMSEA = 0.016, TLI = 0.997, GFI = 0.972, NFI = 0.984, CFI = 0.998, IFI = 0.998. The hostility CFA results were good: χ^2^/*df* = 1.196, RMSEA = 0.022, TLI = 0.993, GFI = 0.957, NFI = 0.974, CFI = 0.996, IFI = 0.996. The smallest item factor loadings was α = 0.461, the highest α = 0.709. A representative item was “Once in a while I can’t control the urge to strike another person.” The questionnaire contains 29 items in total. Items were scored on a five-Likert scale, ranging from 1 = very non-compliant to 5 = very much in line, with higher scores meaning more aggressive behavior. The higher the total score, the higher the level of aggressive behavior in the athlete. The Cronbach’s alpha coefficients for the physical aggression (nine items), verbal aggression (five items), anger (seven items), and hostility (eight items) dimensions were 0.816, 0.755, 0.809, and 0.802, respectively. The Cronbach’s alpha coefficient for the total scale was 0.92 indicating high reliability. The Chinese version of this scale had a Cronbach alpha of 0.94 (e.g., Yang and Wang, 2012).

### Data Analysis

This study used SPSS 21.0 for statistical analysis (including correlation analysis, regression analysis, and variance analysis) and AMOS 20.0 for constructing models and conducting path analyses. Following the two-step procedure recommended by Gerbing and Anderson (1988), this study tested the measurement model before construction. We used CFA for data analysis to confirm the differences between the factors. If the discriminant validity of the variables was good, we would initiate the structural model analysis as the next step.

## Results

### Common Method Bias

There is a risk of common method bias by collecting data using questionnaires; thus, this study adopted the method proposed by previous researchers (Zhou and Long, 2004; Xiong et al., 2012) to control for common method bias. Harman’s single factor test was applied to test for common method bias (Podsakoff et al., 2012). The results showed that there were 16 factors with an eigenvalue greater than 1 and the first factor had an explanatory variance of 21.52%, which is lower than the threshold of 40%, indicating that the common method bias was not significant.

### Self-Efficacy, Self-Control, and Aggressive Behavior: Group Differences

The mean total score for self-efficacy was 3.47, SD = 0.62, indicating that the overall self-efficacy score of this sample of Chinese boxers was generally high. As shown in Table 1, this study found the mean self-efficacy score was significantly different between boxers of different technical grades (*F* = 9.423, *p* < 0.001; i.e., Master or above > Level 3, Level 1 > Level 3, Level 2 > Level 3). A regression analysis found that as the age of the boxers increased, the level of self-efficacy also increased (β = 0.224, *p* < 0.001), and that as the boxers’ number of years of training increased, the level of self-efficacy also increased (β = 0.230, *p* < 0.001).

**Table 1:** Differences in self-efficacy between boxers of different sports levels.

	Level	*M*	SD	*F*	Comparison between groups
Self-efficacy	Master or above	3.75	0.64	9.423^∗∗∗^	Master or above > Level 3, Level 1 > Level 3, Level 2 > Level 3
	Level 1	3.55	0.62		
	Level 2	3.57	0.61		
	Level 3	3.33	0.59		
Self-control	Master or above	3.82	0.48	10.710^∗∗∗^	Master or above > Level 3, Level 1 > Level 3, Level 2 > Level 3
	Level 1	3.76	0.44		
	Level 2	3.88	0.42		
	Level 3	3.57	0.44		


*^∗^ indicates p < 0.05, ^∗∗^ indicates p < 0.01, and ^∗∗∗^ indicates p < 0.001.*

The mean total score for self-control was 3.69, SD = 0.62, indicating that the overall self-control score was generally high. As shown in Table 1, the results show there were significant differences between self-control in boxers belonging to the four different technical grades (*F* = 10.710, *p* < 0.001; i.e., Master or above > Level 3, Level 1 > Level 3, Level 2 > Level 3). A regression analysis found that the higher the age of boxers, the higher the level of self-control (β = 0.188, *p* < 0.001), and that as the boxers’ number of years of training increased, the level of self-control also increased (β = 0.202, *p* < 0.001).

The mean total score for aggressive behavior was 2.23, SD = 0.57, indicating that the overall aggression level of the Chinese boxers was generally low. This study indicates the aggression levels in males and females were significantly different, with males having higher aggression levels than females (*T* = 2.830, *p* < 0.01). Physical aggression levels were significantly different in male and female boxers, with males having higher aggression levels than females (*T* = 3.408, *p* < 0.01). There were significant differences with regard to physical aggression in boxers belonging to the four different technical levels of classification (Master or above, *M* = 1.75, SD = 0.64; Level 1, *M* = 1.94, SD = 0.67; Level 2, *M* = 1.85, SD = 0.66; Level 3, *M* = 2.05, SD = 0.63, *F* = 4.384, *p* < 0.001). A regression analysis found that as the boxers’ number of years of training increased, the lower the physical aggression levels (β = -0.122, *p* < 0.05). Male and female boxers showed significant differences in verbal aggression, with females having higher verbal aggression levels than males (*T* = 2.807, *p* < 0.01). As shown in Table 2.

**Table 2:** Comparison of different gender boxers in Aggressive behavior, Physical aggression, and verbal aggression.

	Gender	*M*	SD	*T*	Comparison between groups
Aggressive behavior	Male	2.32	0.55	2.830^∗^	Male > Female
	Female	2.16	0.58		
Physical aggression	Male	2.08	0.62	3.408^∗^	Male > Female
	Female	1.86	0.66		
Verbal aggression	Male	2.55	0.67	2.807^∗^	Female > Male
	Female	2.75	0.66		


*^∗^ indicates p < 0.05, ^∗∗^ indicates p < 0.01, and ^∗∗∗^ indicates p < 0.001.*

### Self-Efficacy, Self-Control, and Aggressive Behavior: Correlations

As shown in Table 3, self-efficacy was positively correlated with self-control, self-efficacy was negatively related to aggressive behavior, and self-control was correlated negatively with aggressive behavior. The significant correlations between the variables in this study provided a basis for subsequent testing of mediating effects. Therefore, hypotheses H_1_, H_2_, and H_3_ were confirmed.

**Table 3:** Relationships between Demographic Variables, Self-efficacy, Self-control, Aggressive Behavior, and Physical Aggression.

	**M**	SD	1	2	3	4	5	6	7	8	9	10
1. Age	17.72	3.15	–									
2. Number of years of exercise	3.89	2.73	0.836***	–								
3. Competitive level	4.03	1.23	0.720***	0.696***	–							
4. Self-efficacy	3.47	0.62	0.224***	0.230***	0.245***	–						
5. Self-control	3.69	0.46	0.188***	0.202***	0.216***	0.618***	–					
6. Aggressive behavior	2.23	0.57	-0.119*	-0.089	-0.101*	-0.313***	-0.602***	–				
7. Physical aggression	1.95	0.64	-0.148**	-0.122*	-0.159**	-0.230***	-0.522***	0.843***	–			
8. Verbal aggression	2.63	0.74	-0.013	-0.006	0.024	-0.226***	-0.350***	0.749***	0.478***	–		
9. Anger	2.27	0.74	-0.108*	-0.078	0.037	-0.255***	-0.515***	0.844***	0.600***	0.548***	–	
10. Hostility	2.27	0.66	-0.097*	-0.065	0.088	-0.325***	-0.567**	0.866***	0.624***	0.596***	0.631***	–


*^∗^ indicates p < 0.05, ^∗∗^ indicates p < 0.01, and ^∗∗∗^ indicates p < 0.001.*

### The Mediating Effect of Self-Control

Structural equation modeling (SEM) was used to analyze the relationships among the variables with self-efficacy as the exogenous (“independent”) variable and aggressive behavior as the endogenous (“dependent”) variable. Physical aggression, verbal aggression, anger, and hostility were observational variables in the model, and gender was a control variable in the model. According to the testing procedure of mediating effects (Wen et al., 2004; Preacher et al., 2006), the direct effect of self-efficacy on aggressive behavior was to be tested first, followed by the fitness of the model and the significance of each path coefficient after adding in the mediating variable. The fitness indicators of the SEM direct effect analysis results were as follows: χ^2^/*df* = 2.637, RMSEA = 0.063, TLI = 0.967, CFI = 0.989, GFI = 0.992, NFI = 0.983, IFI = 0.989. The direct effect path coefficient of self-efficacy on aggressive behavior was significant (β = -0.36, *p* < 0.001).

**FIGURE 1:**
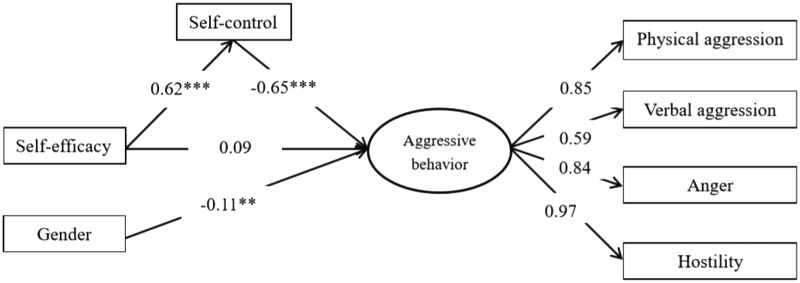
Mediation model of self-control between self-efficacy and aggressive behavior in Chinese boxers (standardized coefficients).

Self-control was placed as a mediating variable between self-efficacy and aggressive behavior in boxers and the fitness results shown in Figure 1 were as follows: χ^2^/*df* = 3.115, RMSEA = 0.072 (90% CI for RMSEA = 0.043, 0.102), TLI = 0.960, CFI = 0.983, GFI = 0.982, NFI = 0.975, IFI = 0.985. The path coefficients between self-efficacy and self-control (β = 0.62, *p* < 0.001), self-control and aggressive behavior (β = -0.65, *p* < 0.001), and gender and aggressive behavior (β = -0.11, *p* < 0.01), were significant. However, after adding the mediating variable, the path coefficient between self-efficacy and aggressive behavior turned from significant (β = -0.36, *p* < 0.001) to non-significant (β = 0.09, SE = 0.043, *p* > 0.05). Therefore, the results indicate that self-control had a full mediating effect on the relationship between self-efficacy and aggressive behavior among boxers, thus confirming hypothesis H_4_ of this study.

## Discussion

The results showed that boxers with high self-efficacy exhibited less aggressive behavior than those with low self-efficacy. Self-control played a mediating role in the relationship between self-efficacy and aggressive behavior in boxers, further revealing the mechanism behind the relationship.

### Group Differences in Self-Efficacy, Self-Control, and Aggressive Behavior

The results of this study indicated that there were significant gender differences in aggressive behavior and physical aggression in boxers, with men showing greater aggressive behavior than women; however, verbal aggression was higher in women than men. Different gender depended to a large extent on differences in individual traits, while it also confirmed prior findings (Coulomb-Cabagno and Rascle, 2006; Maria et al., 2006; Wu and Jiang, 2012). Some scholars believe that there are many reasons for the gender differences in aggressive behaviors, which are the result of the combination of multiple factors (Carol, 1974). Some studies have found that boys are more likely to face risk factors of attack development, such as nervous system dysfunction, difficult temperament, impulsivity, and learning disabilities (Gorman-Smith and Loeber, 2005; Lahey et al., 2006). What’s more, studies have also shown that men are more likely to develop physical aggression due to this different level of emotional arousal (Knight et al., 2010). Men are more aggressive than women in exercise or in daily life (Card et al., 2008; Guerra et al., 2011).

There were no significant gender differences in self-efficacy and self-control. Instead, both the number of years of training and age were significant positive predictors, and the higher the technical grade, the higher the scores on the two variables. These results were in line with previous studies (Vaughn et al., 1984; Zhu and Pan, 2004; Cheng et al., 2010). In the part of demographic difference in self-control and self-efficacy, I have added: as the boxer’s age and the years of exercise increases, the individual’s psychological traits tend to be a process of continuous improvement of mental health; it is obvious that the physical and mental health of boxers have improved a lot, and then the level of self-efficacy and self-control of boxers has increased significantly, which is also in line with the basic laws of human evolution theory.

### Direct Influence of Self-Efficacy on Aggressive Behavior

Correlation analyses showed that self-efficacy was negatively correlated with aggressive behavior in boxers. This is consistent with previous studies (Buser et al., 2015; Brubacher et al., 2016; e.g., Khademi Mofrad and Mehrabi, 2015). Social cognitive theory and self-efficacy theory propose that as self-efficacy increases, aggression levels of individuals decrease, and the expectation value of their own performance and ability rises. This creates a positive attitude in individuals. The results of this study to a certain extent support the views of social cognitive theory and self-efficacy theory, that self-efficacy influences aggression levels in boxers. Most empirical studies have found that self-efficacy can not only affect the individual’s psychological state (Bandura, 1986; Crick and Dodge, 1994) but also effectively deter and reduce the occurrence of aggressive behavior and violence (Caprara et al., 2010). Researchers have also found that among different groups, individuals with high self-efficacy generally exhibited lower aggression levels and were physically and mentally healthier (Brubacher et al., 2016; e.g., Khademi Mofrad and Mehrabi, 2015). With regard to boxing, improving self-efficacy in boxers is not only necessary for performance in competition but also a protective factor in preventing physical and mental health problems. If the level of self-efficacy declines in boxers, it will inevitably increase the likeliness of their aggressive behavior. This study confirms the relationship between self-efficacy and aggressive behavior, whereby self-efficacy is a significant negative predictor of aggressive behavior in boxers.

### Self-Control as a Mediator Between Self-Efficacy and Aggressive Behavior

This study showed that in boxers, self-efficacy was positively correlated with self-control and self-control was negatively correlated with aggressive behavior. This is consistent with previous studies (Hamama and Ronen-shenhav, 2012; Hamedani, 2013; Eingar and Steinhart, 2017). Self-regulation theory and theory of Challenge and Threat States in Athletes propose high self-efficacy and high self-control are important factors for winning at critical moments in sports competitions and they constrain and promote each other (Hamedani, 2013; Li et al., 2013). Meanwhile, this study was also concerned with the deeper mechanisms of self-efficacy and self-control in boxing and offers specific explanations of the theories of self-regulation and challenge and threat states in athletes. Among boxers, self-efficacy and self-control are not only important indicators for selection but also a key factor for competitive performance. Both play a positive role in the development of the physical and mental health of boxers.

In this study, the intrinsic relationship between self-control and aggressive behavior in boxers validates the integrative cognitive model of aggression. Several studies have shown that self-control has the ability to change and constrain aggressive behavior (Denson et al., 2011; Sofia and Cruz, 2015). As a major applied and practical implication, integrating strategies and skills to strengthen self-control capacity and resources (e.g., Duckworth et al., 2014) would certainly be a useful and potential way to tackle the problem of aggression in sport competition, particularly under the stress and pressure situations occurring in sport competition. In intense boxing competitions, boxers who display high self-control are able to reduce impulsivity and maintain rational thinking, which helps them win the game. In addition, if they consider the long-term consequences of their behavior, they will achieve the purpose of reducing aggressive behavior. Therefore, this study establishes the mechanisms behind self-efficacy, self-control, and aggressive behavior.

After confirming the reverse predictive effect of self-efficacy on aggressive behavior, we introduced a mediating variable and explored the mechanism behind the effect. Results indicated that self-control had a full mediating effect in the relationship between self-efficacy and aggressive behavior. From the perspectives of self-regulation theory and the general theory of crime, individuals can rely on their levels of self-control to regulate their own behavior and deter the occurrence of criminal and problem behaviors (Bandura, 2012). By examining the mediating role of self-control in boxers, self-regulation theory and the general theory of crime are further reinforced. We have also found that these two theories are also applicable in boxing. Researchers from different fields have also found that self-control acted as a mediator between other variables and aggressive behavior (Shepperd et al., 2015; Li et al., 2017; e.g., White and Turner, 2014). The same result has been observed in the field of sports (e.g., Zhang, 2016). Combining these results, this study proposes that the self-efficacy should be a focus in competitive training, and that effective training methods should be adopted to improve the ability of self-control, in order to prevent boxers from engaging in aggressive behavior in high-pressure training or competitions. Hence, self-control plays a mediating role between self-efficacy and aggressive behavior in boxers.

### Limitations

Although this study contributes to explore the mechanisms of self-efficacy and aggressive behavior, as well as the mediating role of self-control in that relationship, it has two limitations. Firstly, a cross-sectional design was used in this study, which rendered it impossible to make causal inferences. Future studies should adopt a longitudinal design to better understand the process whereby self-efficacy influence aggressive behavior. Secondly, we focused on the role of self-control in the relationship between self-efficacy and aggressive behavior, but there are still many other important mediating variables to be explored, such as personality traits or self-esteem, which should be also explored in future research. Whether the results of this study can be generalized to other sports remains to be verified. Despite these limitations, the results of this study help us understand the intrinsic relationship between self-efficacy and aggressive behavior to a certain extent as well as its possible causes and mechanisms in the context of Chinese culture. The exploration of the variables and processes involved in this meta-theory, as well as attempted to test its major assumptions, can add a fruitful avenue toward the development and advancement of knowledge about the processes involved in aggression in sport.

## Conclusion

Male displayed more aggressive behavior than female. As age increased, the levels of self-efficacy and self-control also increased. The higher the competitive level, the higher the self-efficacy and self-control; and the higher the number of years of training, the higher the self-efficacy and self-control. Self-efficacy was negatively correlated with aggressive behavior and positively correlated with self-control. Self-control also negatively correlated with aggressive behavior. Furthermore, self-control had a full mediating effect between self-efficacy and aggressive behavior in the current study.

In summary, training boxers’ self-efficacy and self-control in daily training and competition reduces their aggressive behavior. Then, boxers’ self-efficacy, self-control and aggressive behavior should be considered as the key indicator of psychological selection, and the mechanism of psychological selection of boxers should be improved. Therefore, this new conceptual framework can be a valuable and novel perspective for future research of aggression in applied fields such as sport, providing possible targets for intervention and forming a basis for further research.

## Ethics Statement

This study was carried out in accordance with the recommendation of the University’s Human Research Ethics Committee with written informed consent from all subjects. All subjects gave written informed consent in accordance with the Declaration of Helsinki. The protocol was approved by the Southwest University’s Human Research Ethics Committee.

## Author Contributions

XC, GZ, XY, YL, GC, CG-G, and LG conceived the study, interpreted the data, drafted and revised the work, approved the final version of the manuscript to be published, and agreed to be accountable for all aspects of the work.

## Conflict of Interest Statement

The authors declare that the research was conducted in the absence of any commercial or financial relationships that could be construed as a potential conflict of interest.
